# The Distribution of Stroma and Antral Follicles Differs between Insulin-Resistance and Hyperandrogenism-Related Polycystic Ovarian Syndrome

**DOI:** 10.3389/fendo.2017.00117

**Published:** 2017-05-31

**Authors:** Carlo Alviggi, Alessandro Conforti, Pasquale De Rosa, Ida Strina, Stefano Palomba, Roberta Vallone, Salvatore Gizzo, Rosaria Borrelli, Claus Yding Andersen, Giuseppe De Placido, Stefano Guerriero

**Affiliations:** ^1^Department of Neuroscience, Reproductive Medicine, Odontostomatology, University of Naples Federico II, Naples, Italy; ^2^Department of Woman and Child Health, University of Padua, Padua, Italy; ^3^Laboratory of Reproductive Biology, Faculty of Health and Medical Sciences, University Hospital of Copenhagen, Copenhagen, Denmark; ^4^Department of Obstetrics and Gynecology, Policlinico Universitario Duilio Casula, University of Cagliari, Cagliari, Italy

**Keywords:** polycystic ovary syndrome, insulin resistance, ultrasound, hyperandrogenism, polycystic ovary

## Abstract

**Introduction:**

Although insulin resistance plays an important pathogenetic role in polycystic ovary syndrome (PCOS), no correlation between ultrasound PCOS pattern and insulin resistance has yet been reported. The aim of this retrospective observational study was to assess whether the ovarian ultrasonographic parameter differed between PCOS women with insulin resistance and those with a hyperandrogenic profile.

**Materials and methods:**

Women who fulfilled the Rotterdam criteria for PCOS were retrospectively studied. Anthropometric, biochemical, and clinical data were recorded. Women were divided into two groups based on specific transvaginal ultrasound parameters: subjects with more than half of the follicles measuring between 5 and 9 mm in diameter, an ultrasonographic determined stroma/total area (S/A) > 0.34 and a “necklace” sign of antral follicles (Group A); and subjects with more than half of the antral follicles measuring between 2 and 4 mm in diameter, an S/A ≤ 0.34; no “necklace” sign but ubiquitously distributed follicles determined by ultrasound (Group B). The association between these ultrasound patterns and the presence of insulin resistance was also evaluated.

**Results:**

Seventy-eight patients were enrolled: 33 with ultrasound sound pattern A and 45 with pattern B. The latter pattern had a sensitivity of 88% and a specificity of 78% in predicting PCOS women with insulin resistance. There were no differences in age, Ferriman–Gallwey score, and serum gonadotropin or androgen levels between the two groups. Body mass index, the waist-to-hip ratio, and homeostasis model assessment were significantly higher in group B than in group A (*p* < 0.05). Conversely, sex hormone binding globulin levels and ovarian volume were significantly higher in group A (*p* < 0.05). Insulin resistance was more frequent in group B than in group A (36/41, 87.8% versus 7/32, 21.8%; *p* < 0.05).

**Conclusion:**

These results suggest that insulin resistance could be associated with a specific ultrasound pattern in PCOS patients.

## Introduction

Polycystic ovary syndrome (PCOS) is a common endocrine disease affecting approximately 5–10% of women of reproductive age (1). It is clinically characterized by anovulation, oligomenorrhea and hyperandrogenism, and polycystic ovary on ultrasound imaging (2). The ultrasound image of polycystic ovary is typically characterized by an ovarian volume of >10 cm^3^ and/or 12 or more follicles measuring 2–9 mm in diameter. Notably, enlarged ovarian stroma is a marker of PCOS (3, 4). The stroma/total area (S/A) is another diagnostic tool for PCOS; it has a sensitivity of 100% and is highly correlated with plasma androgen level (5).

Insulin resistance is found in a high percentage of women affected by PCOS (6), although neither insulin resistance nor the metabolic syndrome is among the diagnostic criteria for PCOS established by the Rotterdam Consensus Group (7). There is evidence that the pathogenic mechanisms of insulin resistance-related PCOS differ from those underlying hyperandrogenism-related PCOS (6, 8). The diagnostic criteria for insulin resistance are much debated. Currently, the “gold standard” procedure for its diagnosis is the euglycemic clamp and the so-called “minimal model” (9). A less time-consuming method is homeostasis model assessment (HOMA) plus the co-presence of anthropometric alterations [body mass index (BMI) and the waist-to-hip ratio (WHR)] (9). Although insulin resistance plays an important pathogenetic role in PCOS (6), no correlation between ultrasound PCOS pattern and insulin resistance has yet been reported. PCOS women with insulin resistance are usually overweight and can be distinguished from PCOS women with androgenic features, slim appearance, and absence of insulin resistance. These two different “PCOS profiles” are consistent with Rotterdam criteria, however, their physiological and clinical phenotypes are macroscopically different.

The aim of this retrospective observational study was to assess whether the ovarian ultrasonographic parameter differed between PCOS women with insulin resistance and those with a hyperandrogenic profile.

## Materials and Methods

### Patients

We evaluated PCOS patients who fulfilled Rotterdam criteria (7), attending the clinics of Endocrinology of Reproduction and Sterility-Infertility of our Department from January 2013 to December 2015. Considering that there is a dedicated ethic statement below, we could delete this part because it is just a repetition. Anyway feel free to retain it on the basis of journal policy. No additional examination and blood testing were performed except for those required for patients’ assessment. All women signed a consent form for the evaluation of personal data. Inclusion criteria were signs of polycystic ovaries, according to the most recent ESHRE/ASRM consensus criteria (2, 10). Exclusion criteria were as follows: basal follicle-stimulating hormone (FSH) > 10 IU/l; administration of estro-progestin or other hormonal treatment in the previous 6 months; congenital adrenal hyperplasia and other endocrine abnormalities; presence of ovarian formations with diameters > 14 mm in two ultrasound examinations carried at an interval of 30 days; thyroid disorders; diabetes mellitus; presence of a single ovary; and previous ovary surgery.

The patients’ anthropometric characteristics (weight, height, BMI, and WHR) were collected as well as serum levels of gonadotropins, DHEA-S, and free testosterone measured in the early follicular phase. We also evaluated hirsutism with the Ferriman–Gallwey clinical score. All enrolled subjects underwent a 75-g oral glucose challenge and insulin measurements at baseline and 60, 120, and 180 min thereafter. We adopted strict criteria to define the “presence” or “absence” of ultrasonographic patterns related to insulin resistance. Thus, women were included in Group 1 (insulin resistance) only when all the following criteria were fulfilled: HOMA > 2.5 (9); BMI > 27 kg/m^2^; WHR > 0.85 (11); sex hormone binding globulin (SHBG) serum levels below the 25th percentile (9, 11); and serum insulin > 150 IU/ml at 60 min and a change of <20% between 60 and 120 min after oral glucose load with 75 g of glucose (12). Similarly, only women whose values were within normal range served as control group [no-insulin resistance (Group 2)]. Consequently, women with an intermediate condition, namely those not showing the copresence or coabsence of all the aforementioned criteria were excluded from the study. In fact, taken separately, they could not be sufficiently specific to identify women with insulin resistance.

### Ultrasound Parameters

**Figure 1:**
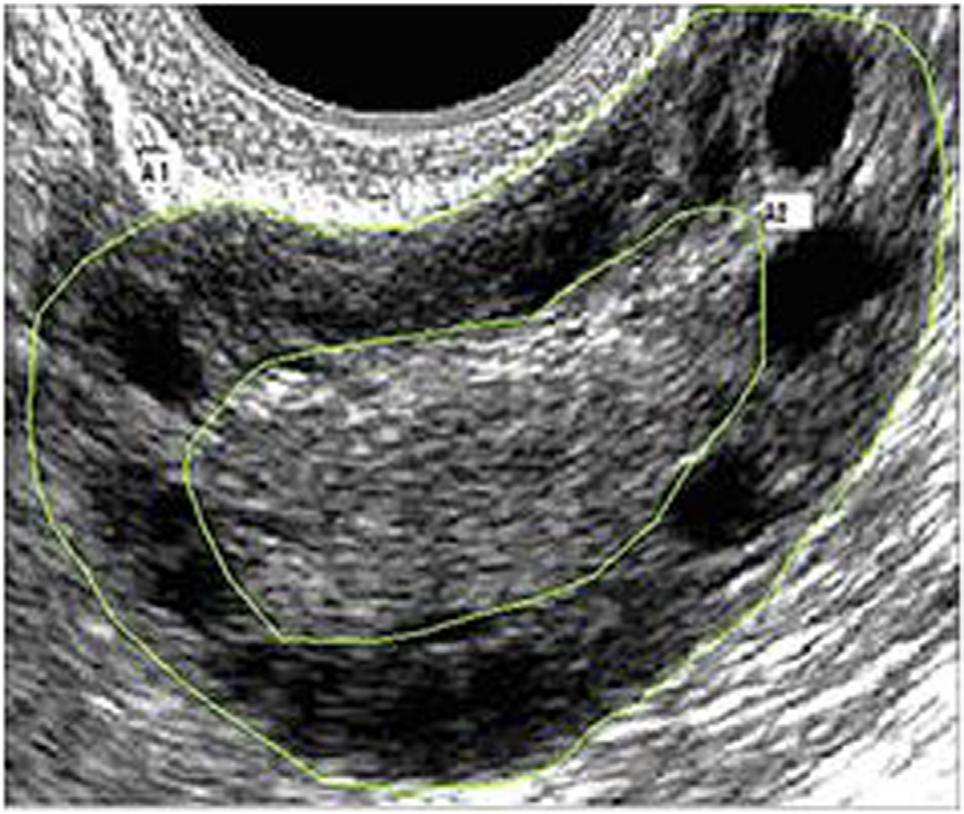
**Example of median ovarian section with the ovarian and stromal total areas defined**. Calipers are positioned so as to encircle the total gonad circumference (A1) and the stromal component circumference (A2). The stroma/total area was also calculated.

**Figure 2:**
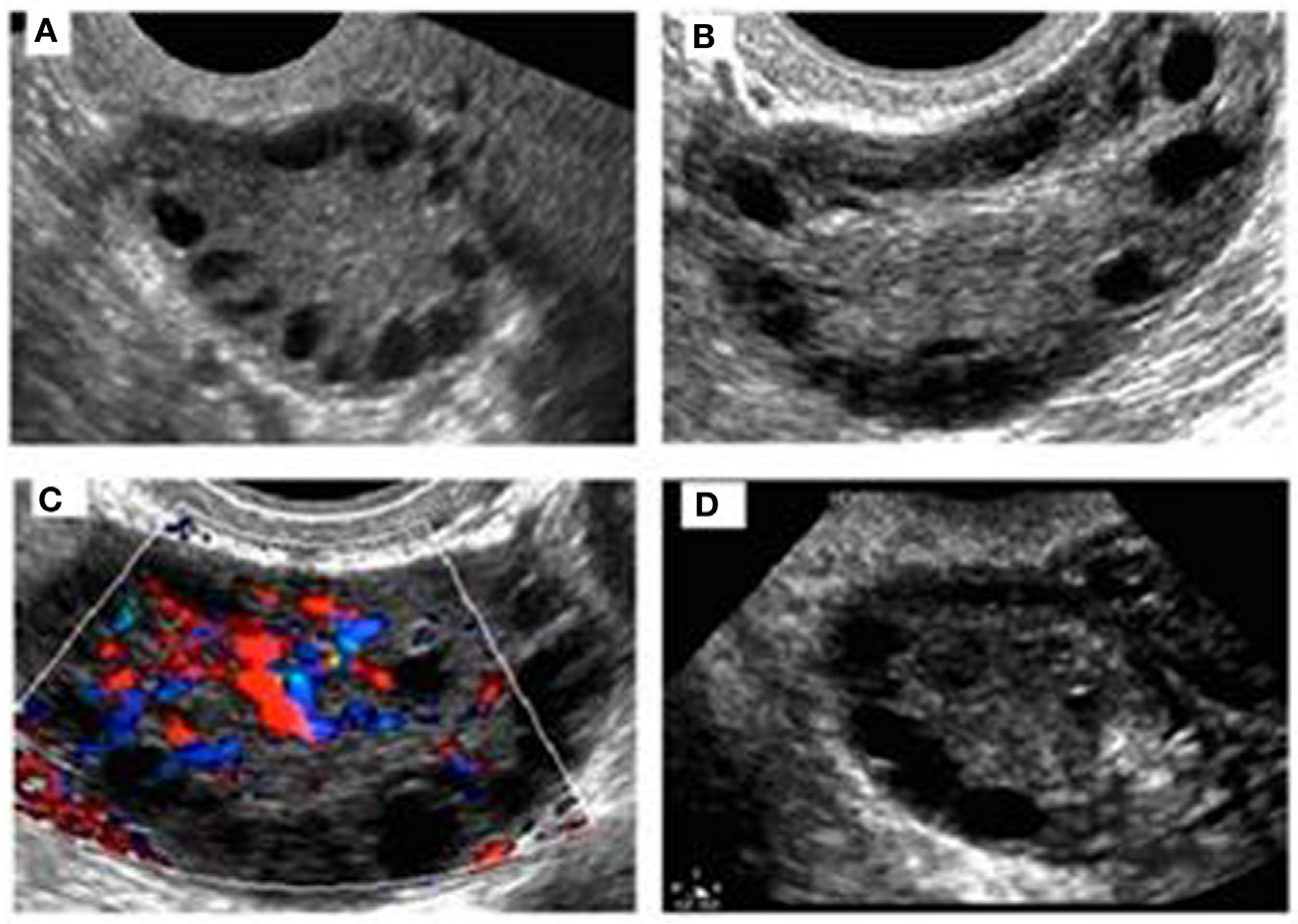
**Sonographic pattern of a type A ovary (A)**. Note the typical rosary arrangement of follicles and the easily recognizable hyperechogenicity that results from thickening of theca **(B)**. The stromal hypervascularity is clearly visible in **(C)**. Secondary aspects are the dominance of follicles with a diameter of >4 mm and the predominance of longitudinal diameter **(D)**.

**Figure 3:**
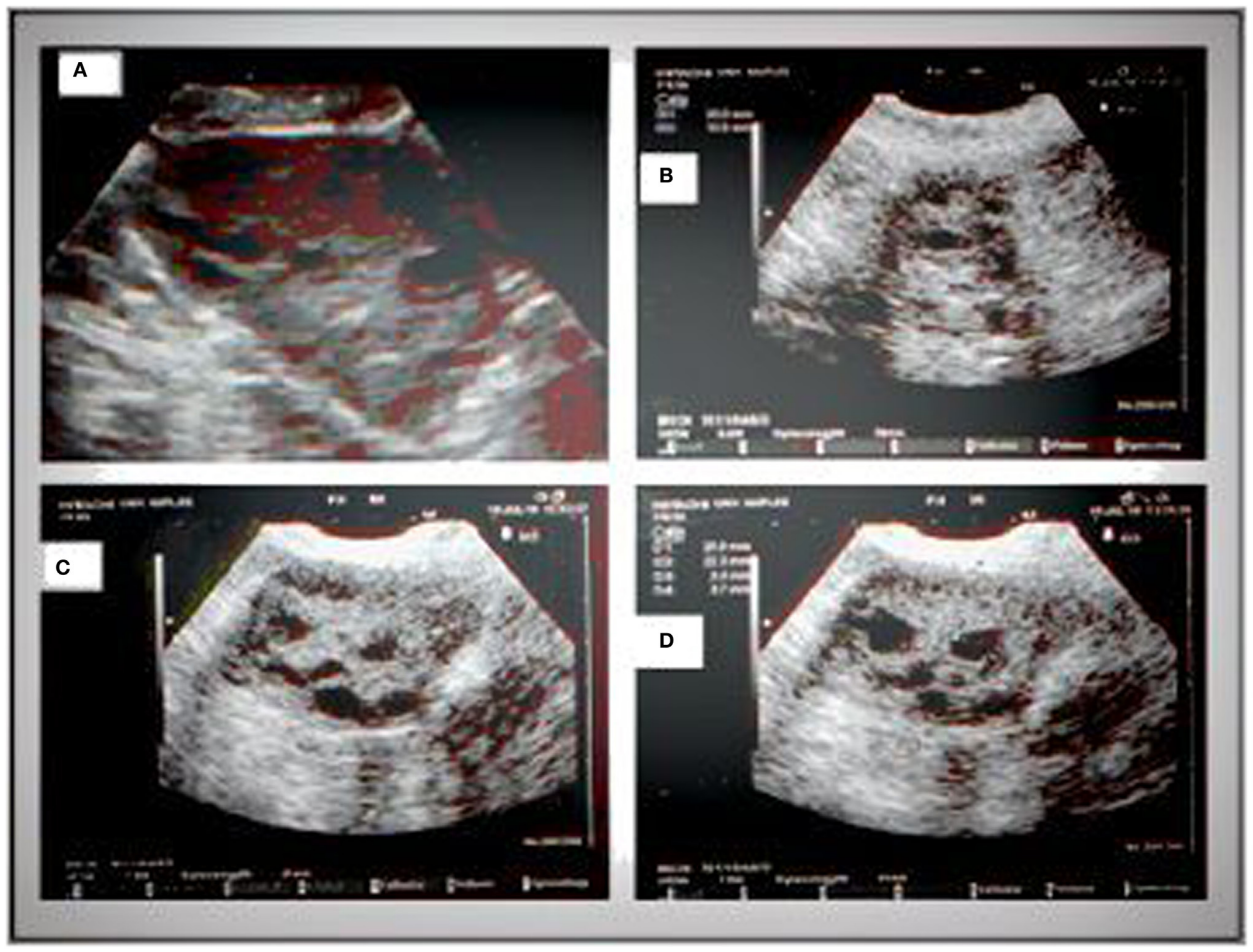
**Sonographic pattern of a type B ovary (A)**. Note the ubiquitous arrangement of follicles and the absence of central echogenicity **(B)**. Characteristic signs of the type B ovary are a more “globular” gonad versus a type A ovary **(C)**, with attenuation of the typical dominance of longitudinal diameter and the presence of follicles with a mean diameter lower than those observed in type A **(D)**.

Pelvic ultrasound examination was carried out with a 6.5 MHz vaginal probe. The external circumferences of the ovary and the stroma were measured to determine the S/A ratio (Figure 1). The following sonographic parameters were recorded for each patient: total number of ovarian follicles with a diameter < 10 mm; number of follicles with diameter between 2 and 4 mm; the S/A ratio calculated with an ovarian median scan (5); and ovarian longitudinal (A), transverse (B), and coronal (C) diameters and volume [1/2 × (A × B × C)] (13). Based on these parameters, we arbitrarily defined two ultrasound patterns (Figures 2 and 3): type A (Group A) characterized by >50% follicles with diameters between 5 and 9 mm and with an S/A > 0.34 (“necklace” sign defined by a hyperechoic central area and a rosary-like peripheral disposition of follicles) and type B (Group B) characterized by >50% follicles measuring 2–4 mm and with an S/A ≤ 0.34 (no “necklace” sign and ubiquitously distributed follicles). Intermediate ultrasound patterns were not included in our analysis.

### Statistical Analysis

Results are reported as mean ± SD. The Student’s *t*-test or the Mann–Whitney *U* test was used for continuous variables with a parametric or no parametric distribution, respectively. Normal distribution of continuous variables was evaluated with the Shapiro test. Cohen’s kappa was used to evaluate the agreement between sonographers with respect to ultrasound ovarian pattern. Sensitivity, specificity, positive predictive value, negative predictive value, and positive and negative likelihood ratio were calculated with the MedCalc statistic software to assess the accuracy of an ultrasound pattern in identifying PCOS patients with insulin resistance. The χ^2^ test was used to compare categorical data. A *p* < 0.05 was considered statistically significant. The SPSS, statistical software 18.0 (SPSS Inc., USA) was used to analyze data.

## Results

**Figure 4:**
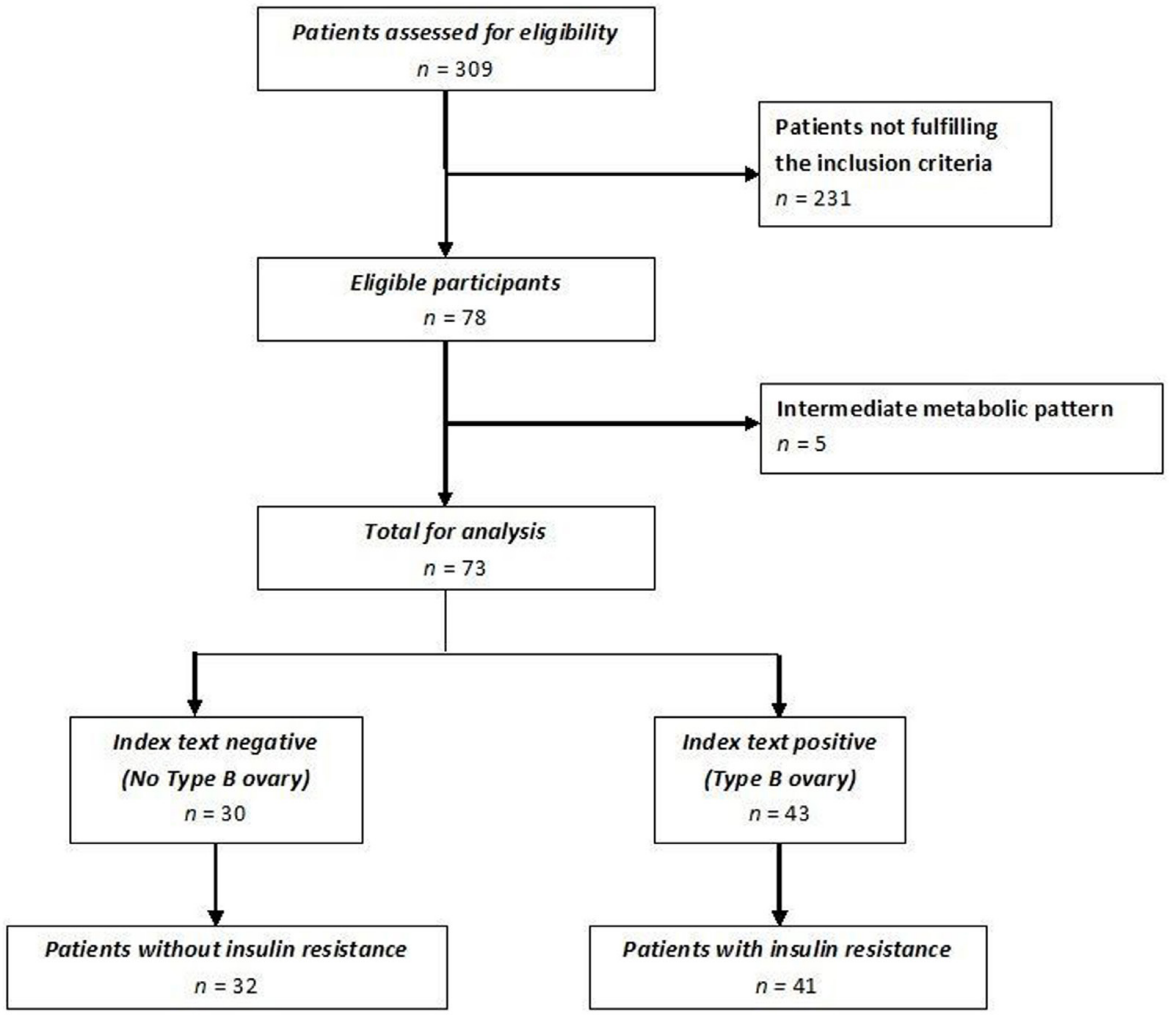
**Flow chart of patients enrolled in the study Standards for Reporting Diagnostic Accuracy**.

Of the 309 patients affected by PCOS admitted to our institute, only women without an intermediate ultrasound profile and fulfilling inclusion and exclusion criteria were included for a total of 78 patients enrolled (Figure 4). Forty-one of these women met all the criteria of insulin resistance (Group 1); the remaining 32 patients did not fulfill any insulin resistance criterion (Group 2). Five women with an intermediate metabolic profile were not included in our analysis (Figure 4). Demographic, anthropometric, hormonal, and ultrasound features of the two groups stratified according to insulin resistance are listed in Table 1. There was no difference in terms of age, androgen levels, or Ferriman–Gallwey score between the two groups. Women affected by insulin resistance were more likely to have a type B ultrasound pattern than women without insulin resistance (87.8 versus 21.8%, *p* < 0.01).

**Table 1:** **Demographic, anthropometric, and hormonal characteristics, and ultrasound pattern frequency in the study population stratified according to insulin resistance**.

Characteristics	Group 1 (*n* = 41)	Group 2 (*n* = 32)	*p*-Value
Age (years)	28.6 ± 6	28.5 ± 4.8	0.93
BMI (kg/m^2^)	34.5 ± 5.9	23.5 ± 2.5	0.0001
WHR	0.9 ± 0.5	0.8 ± 0.8	0.0001
HOMA	5.1 ± 2.9	1.5 ± 0.6	0.0001
Type B ovary	36/41 (87.8%)	7/32 (21.8%)	0.0001
Type A ovary	5/41 (12.1%)	25/32 (78.1%)	0.0001
Ovarian volume (cm^3^)	14.6 ± 5.6	11.9 ± 4.1	0.024
SHBG (nmol/l)	27.2 ± 11.3	52.2 ± 21.1	0.0001
Ferriman–Gallwey score	10.7 ± 3.3	11.3 ± 3.2	0.47
Free testosterone (pg/ml)	2.5 ± 2.6	1.9 ± 1.5	0.36
DHEA-S (μg/dl)	282.1 ± 391.1	252.5 ± 304	0.74
17-OH-P (ng/ml)	1.2 ± 0.9	1.9 ± 1.7	0.93

*Results reported as mean ± SD or percentage (%)*.

*BMI, body mass index; WHR, waist-to-hip ratio; HOMA, homeostasis model assessment; SHBG, sex hormone binding globulin; 17-OH-P, 17-hydroxy progesterone*.

The accuracy of the type B ultrasound pattern to identify PCOS women with insulin resistance was as follows: sensitivity 0.88 CI 95% (0.74–0.96); specificity 0.78 CI 95% (0.60–0.91), positive likelihood ratio 4.01 CI 95% (2.06–7.80), negative likelihood ratio 0.16 CI 95% (0.07–0.36); and positive predictive value 0.84 CI 95% (0.69–93.2), negative predictive value 0.83 CI 95% (0.65–0.94). A Cohen’s kappa of 0.81, estimated in 50 subjects, indicated an excellent concordance between sonographers (14).

The demographic, anthropometric, and hormonal data of patients divided according to the ultrasound pattern of PCOS, including also five women with intermediate metabolic condition, are listed in Table 2. The mean age of patients, serum levels of gonadotropin, androgens, and the Ferriman–Gallwey score did not differ significantly between the two groups. Conversely, BMI, WHR, and HOMA values were significantly higher in patients with a B-type ultrasound pattern. Patients with an A-type pattern had higher SHBG levels (27.2 ± 11.3 nmol/l versus 52.2 ± 21.1 nmol/l, *p* < 0.01) and a larger ovarian volume (14.6 ± 5.6 versus 11.9 ± 4.1 cm^3^, *p* < 0.05) than did patients with an A-type pattern.

**Table 2:** **Demographic, anthropometric, and hormonal characteristics of patients divided according to the ultrasound pattern of PCOS**.

Characteristics	Group A (type A ovary) (*n* = 33)	Group B (type B ovary) (*n* = 45)	*p*-Value
Age (years)	28.4 ± 5.4	29 ± 5.8	0.61
BMI (kg/m^2^)	23.7 ± 2.3	43.1 ± 56	0.048
WHR	0.81 ± 0.6	0.92 ± 0.5	0.0001
HOMA	1.5 ± 0.8	4.9 ± 2.8	0.0001
LH (UI/l)	9.25 ± 6.3	5.2 ± 3.3	0.002
Ovary volume (cm^3^)	12.1 ± 4	14.3 ± 5.6	0.048
SHBG (nmol/l)	53.5 ± 23.5	28 ± 11.6	0.0001
Ferriman–Gallwey	11.6 ± 2.9	10.5 ± 3.4	0.14
Free testosterone (pg/ml)	2.6 ± 2.6	2.1 ± 1.7	0.27
DHEA-S (μg/dl)	224.7 ± 221.7	164.4 ± 109.2	0.14
17-OH-P (ng/ml)	1.8 ± 1.5	1.2 ± 1.3	0.057

*Results are reported as mean ± SD*.

*BMI, body mass index; WHR, waist-to-hip ratio; HOMA, homeostasis model assessment; PCOM, polycystic ovarian morphology; LH, luteinizing hormone; SHBG, sex hormone binding globulin; 17-OH-P, 17-hydroxy progesterone*.

## Discussion

We have identified an association between metabolic parameters and ultrasound pattern in patients affected by PCOS. BMI, WHR, and HOMA were significantly lower in patients with a type A ovary, namely those with the classical “necklace” sign than in women with a type B ovary. The association of a high HOMA with elevated anthropometric indices was previously reported to be an efficient positive predictor of insulin resistance (9). The association between insulin resistance and specific ultrasound patterns is supported by data obtained when we stratified our study population into patients with insulin resistance (Group 1) and patients without insulin resistance (Group 2). Type B ovaries were significantly more frequent in group 1 than in group 2. On the contrary, the classic ultrasound picture of PCOS (type A) was more frequent in Group 2 patients. Neither serum concentrations of androgens nor the Ferriman–Gallwey score differed between the two groups. However, all biochemical and clinical androgenic variables, including basal luteinizing hormone (LH), were higher in patients with a type A ovary than in those with a type B ovary, and the difference was almost significant in the case of 17-OH P concentrations (Table 2).

The different ovarian profiles observed in our study support the concept that the pathogenesis and clinical phenotype could differ between PCOS patients with hyperandrogenism and normal anthropometric parameters and PCOS patients with insulin resistance. In other words, two physiopathogenetic pathways, one characterized by hyperandrogenism and the other by insulin resistance, could induce the same effects, namely, they could interfere with selection mechanisms of the dominant follicle and also induce atresia of secondary follicles. These changes in folliculogenesis could result in anovulation, arrest of multiple follicles at different developmental stages, and hyperandrogenism, which could be either primitive or secondary depending on clinical conditions.

Based on our preliminary data, the classical ultrasound imaging characterized by a hyperechoic central area and a peripheral “necklace” arrangement of follicles is more typically observed in PCOS patients who have a more pronounced hyperandrogenic profile, minimal or absent insulin resistance, and pronounced hypertrichosis.

Ovarian morphology changes dramatically should the *primum movens* of PCOS development be insulin resistance. Normally, insulin, by way of the classic mechanism of “spill-over,” binds insulin growth factor-1 (IGF-1) receptor thereby exerting mitogenic effects on the granulosa and theca. IGF-2 plays a pivotal role in the FSH-mediated proliferation of the granulosa and is hence important for the growth and development of follicles (15). If the proliferation of antral follicles is not finely regulated, the mechanisms governing the selection of the dominant follicle could be deranged thereby fostering follicle atresia. Our data suggest that the growth of antral follicles is blocked before in PCOS women with insulin resistance. In fact, type B ovaries are characterized by a predominance of follicles with a diameter measuring between 2 and 4 mm. In addition, lack of prominent hyperandrogenism could explain absence of hyperthecosis, which in turn may lead to a ubiquitous distribution of follicles. Although insulin production could reduce SHBG concentrations and so lead to an increase in the free forms of various androgens particularly testosterone (2), this effect does not seem sufficient to induce the hyperthecosis typical of PCOS associated with a hyperandrogenic pattern. Our results support this hypothesis. In fact, androgen levels and clinical hyperandrogenism indices were lower in our patients with a type B ovary and partially in the hyperinsulinemic patients (Tables 1 and 2).

The hypothesis that different PCOS profiles have specific physiopathological pathways may have important implications for the management of patients and might imply a revision of the current PCOS diagnostic criteria. In other words, the attempt of the Rotterdam Consensus Group (7) to define a shared, universal diagnosis of PCOS by gathering together patients with different characteristics and phenotypes may lead to a suboptimal approach for these patients even in fertility management. In detail, the insulinemic pattern seems to significantly influence ovarian response to gonadotropin administration (16) and ovarian drilling success (17). An increased risk of ovarian hyperstimulation syndrome in hyperinsulinemic patients has also been reported (18). The insulin profile may also indirectly affect ovarian stimulation. In fact, insulin-sensitizing agents such as metformin before and during IVF/ICSI significantly reduced the incidence of ovarian hyperstimulation and improved the pregnancy rate (19). On the other hand, a hyperandrogenic profile may impair AMH production and lead to dysfunction of folliculogenesis (20). The higher basal LH levels usually observed in hyperandrogenic women could also influence the ovarian response (21–23). In detail, PCOS patients with an elevated LH/FSH ratio had a better pregnancy rate when treated with GnRH agonist protocols than with GnRH antagonist protocols, probably because the long GnRH agonist induces prolonged LH suppression milieu thereby avoiding the negative effect of higher LH levels on reproductive outcome (21, 23, 24). Consequently, strategies that minimize the effect of LH could be considered for patients with a hyperandrogenic profile.

A possible limitation of this study is the lack of a control population. Initially, we intended to focus on the heterogeneity within PCOS women according to Rotterdam criteria. Following confirmation of these preliminary observations, larger studies that include a healthy control population would be necessary to better define differences among groups. Another limit is the retrospective design *per se*. Should our preliminary observations be confirmed in a prospective trial including confirmation of inter-reliability among operators, all the ultrasonographic features should be analyzed separately in order to identify the features most predictive of the insulin resistance.

In conclusion, we found that insulin resistance in PCOS patients is associated with a peculiar ultrasound pattern. This observation, if confirmed by larger studies, supports the concept that specific PCOS profiles could be identified by a complete metabolic evaluation and targeted ultrasound evaluation. Given the paucity of data regarding this issue, it remains to be seen if different PCOS profiles will lead to different diagnostic and eventually more tailored therapeutic approaches.

## Ethics Statement

Given the observational and retrospective no pharmacological character of this study, Ethics Committee approval was not required. No additional examination and blood testing were performed except for those required for patients’ assessment. All women signed a consent form for the evaluation of personal data.

## Author Contributions

All the authors have made substantial, direct, and intellectual contribution to the work and approved it for publication.

## Conflict of Interest Statement

The authors declare that the research was conducted in the absence of any commercial or financial relationships that could be construed as a potential conflict of interest.
